# Prenatal THC Exposure Induces Sex-Dependent Neuropsychiatric Endophenotypes in Offspring and Long-Term Disruptions in Fatty-Acid Signaling Pathways Directly in the Mesolimbic Circuitry

**DOI:** 10.1523/ENEURO.0253-22.2022

**Published:** 2022-10-07

**Authors:** Mohammed H. Sarikahya, Samantha Cousineau, Marta De Felice, Kendrick Lee, Karen KW Wong, Marieka V. DeVuono, Tony Jung, Mar Rodríguez-Ruiz, Tsun Hay Jason Ng, Dana Gummerson, Emma Proud, Daniel B. Hardy, Ken K.-C. Yeung, Walter Rushlow, Steven R. Laviolette

**Affiliations:** 1 Addiction Research Group, Schulich School of Medicine and Dentistry, Western University, London, Ontario N6A 5C1, Canada; 2 Department of Anatomy and Cell Biology, Western University, London, Ontario N6A 3K7, Canada; 3 St. Josephs Health Care, Lawson Health Research Institute, London, Ontario N6C 2R5, Canada; 4 Departments of Biochemistry and Chemistry, Western University, London, Ontario N6A 5C1, Canada; 5 Departments of Physiology and Pharmacology and Obstetrics and Gynaecology, Western University, London, Ontario N6A 5C1, Canada; 6 St. Josephs Health Care, Children’s Health Research Institute, London, Ontario N6C 2R5, Canada; 7 Department of Psychiatry, Western University, London, Ontario N6A 3K7, Canada

**Keywords:** electrophysiology, lipidomics, MALDI IMS, mesolimbic system, prenatal cannabis exposure, sex differences

## Abstract

Despite increased prevalence of maternal cannabis use, little is understood regarding potential long-term effects of prenatal cannabis exposure (PCE) on neurodevelopmental outcomes. While neurodevelopmental cannabis exposure increases the risk of developing affective/mood disorders in adulthood, the precise neuropathophysiological mechanisms in male and female offspring are largely unknown. Given the interconnectivity of the endocannabinoid (ECb) system and the brain’s fatty acid pathways, we hypothesized that prenatal exposure to Δ^9^-tetrahydrocannabinol (THC) may dysregulate fetal neurodevelopment through alterations of fatty-acid dependent synaptic and neuronal function in the mesolimbic system. To investigate this, pregnant Wistar rats were exposed to vehicle or THC (3 mg/kg) from gestational day (GD)7 until GD22. Anxiety-like, depressive-like, and reward-seeking behavior, electrophysiology, and molecular assays were performed on adult male/female offspring. Imaging of fatty acids using matrix-assisted laser desorption/ionization imaging mass spectrometry (MALDI IMS) was performed at prepubescence and adulthood. We report that PCE induces behavioral, neuronal, and molecular alterations in the mesolimbic system in male and female offspring, resembling neuropsychiatric endophenotypes. Additionally, PCE resulted in profound dysregulation of critical fatty acid pathways in the developing brain lipidome. Female progeny exhibited significant alterations to fatty acid levels at prepubescence but recovered from these deficits by early adulthood. In contrast, males exhibited persistent fatty acid deficits into adulthood. Moreover, both sexes maintained enduring abnormalities in glutamatergic/GABAergic function in the nucleus accumbens (NAc). These findings identify several novel long-term risks of maternal cannabis use and demonstrate for the first time, sex-related effects of maternal cannabinoid exposure directly in the developing neural lipidome.

## Significance Statement

Prenatal cannabinoid exposure (PCE) is growing in prevalence and the long-term effects on fetal brain development are largely unknown. Our study provides novel evidence that chronic prenatal Δ^9^-tetrahydrocannabinol (THC) exposure can induce long-lasting alterations in mesolimbic function that differentially impacts the male versus female brains. We also demonstrate for the first time that PCE can induce profound lipidomic adaptations that may account for these neuropathophysiological sequelae.

## Introduction

With increasing global legalization, cannabis use during pregnancy is rising considerably with the accompanying perception that it may serve as a natural therapeutic for pregnancy-related nausea, depression, and/or anxiety (Brown et al., 2019; Roncero et al., 2020). However, growing evidence suggests that prenatal cannabis exposure (PCE), particularly to its main psychotropic component, Δ^9^-tetrahydrocannabinol (THC), enhances the risk for childhood and later-life psychopathology (Patel et al., 2020). While the precise neurobiological mechanisms in both male and female offspring remain poorly understood, it is well established that THC disrupts neurodevelopmental endocannabinoid (ECb) signaling pathways, which in turn modulate various fatty acid pathways critical for early synaptic development and functional integrity (Dyall, 2017; Freitas et al., 2018; Watson et al., 2019).

During prenatal brain development, the ECb system is involved in regulating various neurodevelopmental processes through the spatiotemporal engagement of the cannabinoid receptor 1 (CB1R; Castillo et al., 2012; Lu and Mackie, 2021). CB1Rs are highly expressed in the mesolimbic system (Lu and Mackie, 2021) and are thus particularly vulnerable to the effects of THC (Fitoussi et al., 2018). The mesolimbic DA pathway, projecting from the ventral tegmental area (VTA) to the nucleus accumbens (NAc), is involved in affective regulation, reward processing, and anxiety-related phenomena (Berridge, 2012; Rey et al., 2012). Given the ECb systems role in neuronal growth and synaptic function (Berghuis et al., 2007; Lu and Mackie, 2021), disruptions induced by THC could conceivably cause aberrations in the development of mesolimbic neuronal circuits (Frau et al., 2019; Scheyer et al., 2019; Sagheddu et al., 2021; Traccis et al., 2021).

Interestingly, two key components of neural and synaptic membranes, composing up to 60% of neuronal membranes, the polyunsaturated fatty acids (PUFA), docosahexaenoic acid (DHA; omega−3), and arachidonic acid (ARA; omega−6) are functionally interdependent with the ECb system (Davis-Bruno and Tassinari, 2011; Lauritzen et al., 2016). Both PUFAs are primarily acquired through the maternal diet during gestation as precursors or in esterified form (Davis-Bruno and Tassinari, 2011; Heath et al., 2022). PUFA precursor conversion is dependent on hepatic CB1R-mediated enzymatic machinery (Lamaziere et al., 2013; Watson et al., 2019). Once trafficked to the brain, both DHA/ARA can be converted into the primary operational ECb agonists [e.g., anandamide, 2-arachidonyl glycerol (2-AG), and docosahexaenoyl ethanolamide (DHEA); Watson et al., 2019; Bakker, 2020; Lu and Mackie, 2021]. These essential fatty acids are also components of neural plasma membranes and are necessary for numerous neurodevelopmental processes (Davis-Bruno and Tassinari, 2011; Hishikawa et al., 2017). DHA and ARA play significant roles in dopamine (DA), glutamate (GLUT), and GABAergic neurotransmission within the mesolimbic circuitry (Vancassel et al., 2007; Lauritzen et al., 2016). Consequently, gestational dietary DHA and ARA restriction adversely impact neural function and result in enhanced anxiety and deficits in emotional processing in rodents (McNamara and Carlson, 2006; Wood et al., 2014; Maekawa et al., 2017).

Given the relationships between ECb and fatty acid signaling regulation, we hypothesized that PCE may induce a sex-specific (Gillies et al., 2020; Lee and Hardy, 2021) neuropsychiatric-like phenotype in offspring that is mediated by fatty acid signaling dysregulation. To describe the long-term impacts of PCE, in a clinically relevant THC dose, we assessed postnatal pathophenotypes using a well-established rodent model of prenatal THC exposure (3 mg/kg) in Wistar rat dams (Gillies et al., 2020; Natale et al., 2020; Lee and Hardy, 2021; K Lee et al., 2021). We report that maternal THC exposure induces long-lasting and highly sex-specific anxiogenic behavioral phenotypes, neuronal dysregulation in the VTA, and disruptions in DA, GLUT, and GABA molecular biomarkers. Importantly, we identify for the first time profound and sex-specific THC-induced effects on the developing mesolimbic lipidome, which may underlie the persistent pathophysiological effects of maternal cannabinoid exposure in offspring.

## Materials and Methods

Behavioral experiments were conducted on male and female progeny between postnatal day (PD)70 and PD100, electrophysiology between PD100 and PD120, and protein quantification and fatty acid analyses, using matrix-assisted laser desorption/ionization imaging mass spectrometry (MALDI IMS), on PD21 and PD120. 1,6-diphenyl-1,3,5-hexatriene (DPH) was selected for the MALDI IMS matrix to assess fatty acids present in tissue (Ibrahim et al., 2017). Adult female estrous cycles were recorded on each experiment day.

### Animals and drug treatments

All procedures were performed according to guidelines set by the Canadian Council of Animal Care, and Animal Use Protocol (#2019-126 for D.H.B. and #2018-056 for S.R.L.) was approved by the Animal Care Committee at Western University. All investigators understood and followed the ethical principles outlined by Grundy (2015), and study design was informed by ARRIVE guidelines (Kilkenny et al., 2010). Pregnant Wistar rats (*n* = 25, 200–254 g) arrived on gestational day (GD)3 from Charles River Canada (Quebec) and were maintained at 22°C on a 12/12 h light/dark cycle with access to food and water *ad libitum*. Dams were randomly assigned to either vehicle (VEH; *n* = 12) or 3 mg/kg THC (*n* = 13) daily via intraperitoneal injection from GD7 to GD22. Litter size was not significantly affected (*n* = 11–14 pups/dam), consistent with previous publications using this model (Gillies et al., 2020; Natale et al., 2020; K Lee et al., 2021). Birth weight was significantly affected with the THC exposed group exhibiting significantly lower birth weight than VEH, consistent with previous publications using this model (Gillies et al., 2020; Natale et al., 2020; K Lee et al., 2021). Litter size was limited to *n* = 8 to ensure equalization of postnatal nutrition. Per dam, four male and four female offspring were kept, the remaining offspring were killed on PD1 (parturition). Male and female offspring were weaned on PD21 and litter-housed, until exceeding institutional cage weight guidelines, and then pair-housed with litter until PD120. Male and female progeny were placed in separate rooms. Between *n* = 1–2 male and female progeny from each dam was killed at PD21 and the remaining were killed at PD120, to ensure that there were no outlier dams or cohort effects that may instead account for offspring outcomes at each developmental endpoint. The open field test (OFT), sucrose preference test, and conditioned place preference (CPP) test were conducted on one cohort (*n* = 12/treatment/sex), with *n* = 6/treatment/sex used for electrophysiology and *n* = 6/treatment/sex used for protein quantification. EPM and LDB were conducted on a second cohort of offspring (male: *n* = 17/treatment; female: *n* = 20/treatment), and were then used for protein quantification, MALDI IMS, and electrophysiology.

### Behavioral testing

Behavioral phenotypes were assessed during adulthood beginning on PD70 and ending on PD100 in males and females. Between experiments, rats were returned to their home cage for a minimum of 24 h. The behavioral assays used were the (1) conditioned place preference test, (2) elevated plus maze (EPM), (3) light/dark test, (4) open field test, and (5) sucrose preference test.

### Subthreshold morphine conditioned place preference test

The subthreshold morphine conditioned place preference test was conducted as previously described (Norris et al., 2019). We have previously reported that this subreward threshold conditioning dose of morphine (0.05 mg/kg, i.p.) is exquisitely sensitive to interventions that increase opioid reward sensitivity (20), serving as a highly effective behavioral conditioning assay in rats to interrogate systemic alterations in opioid reward sensitivity. Rats were conditioned using an unbiased counterbalanced place conditioning procedure. The two conditioning environments differed in smell, texture and color. One environment was black, with a Plexiglas floor, wiped down with 2% acetic acid, to ensure novelty of context (smell), before each conditioning stage. The second environment was white, with a wire mesh floor covered with woodchips. Before CPP, 24 h before the start of conditioning, rats were preconditioned, where they are placed into a motivationally neutral gray box for 20 min. CPP conditioning consists of four drug-environment and four vehicle-environment pairings once per day for 30 min each session, alternating over an 8-d period. Environmental conditioning exposures are fully counterbalanced for both environment assignment and drug/vehicle presentations. During the CPP test phase, rats are placed in a neutral gray zone separating the drug and vehicle environments and allowed to move freely for a period of 10 min between environments. One week after the conditioning phase, in a drug-free state, the rats were tested. Times spent in each environment were digitally recorded and analyzed offline.

### Elevated plus maze

The elevated plus (EPM) is a measure of anxiety. The assessment was conducted as described elsewhere (Renard et al., 2017, 2018; Szkudlarek et al., 2019). The EPM apparatus is made of black acrylic with four arms (10 × 50 cm), on a raised platform (50 cm), illuminated at 40 lux. Two arms opposite each other are enclosed with 40 cm high acrylic walls, while the other two opposing arms are opened, with a 1-cm-high barrier. Rats are placed in the center of the apparatus facing a closed arm and were allowed 10 min to explore the maze. Anxiety-like behavior was measured as the number of entries (i.e., all four paws in arm) and time spent in closed and open arms. Behavior was video recorded and analyzed offline (Behaview software).

### Light dark box test

The light-dark box test is a measure for anxiety. The assessment was conducted as described elsewhere (Renard et al., 2017, 2018; Szkudlarek et al., 2019). The apparatus consists of two (50 × 25 × 37 cm) Plexiglas compartments divided by a wall with a small opening (10 × 10 cm). The “dark” side consists of black walls and a lid to prevent any light entering the compartment. The “light” side consists of white walls with an open top and an overhead 1500 lux light. The rat is placed within the light side of the apparatus and allowed to freely explore both compartments for 10 min. Anxiety-like behavior was measured based on the total number of transitions between the light and dark compartments, the total percent time spent in the light compartments, and the latency to transition back from the dark to light environment (i.e., second transition latency). Behavior was video recorded and analyzed offline (Behaview software).

### Open field test

The open field test functions as both a measure of anxiety and motility. The assessment was conducted as described elsewhere (Renard et al., 2017, 2018; Szkudlarek et al., 2019). Rats were placed in an automated open field activity chamber (Med Associates) for 30 min. The open field apparatus consists of a clear acrylic chamber (80 × 80 × 50 cm) brightly illuminated (300 lux in center). Rats, naive to the apparatus, are placed in the center of the apparatus and allowed to explore for 10 min. Motility was assessed as the total distance traveled during the 10 min, while anxiety was assessed as number of entries to the center portion of the apparatus during the first 5 min of the test (thigmotaxis).

### Sucrose preference test

The sucrose preference test is commonly used to assess anhedonia. Rats received *ad libitum* access to a 2% sucrose solution in their home cages, without access to water, to allow for acclimation to a palatable liquid-sucrose solution. Rats were then deprived of all fluids for 12 h before testing. On test day, rats were given *ad libitum* access to two bottles, one containing water and one with a 2% sucrose solution. Fluid intake volume was normalized to body weight. Sucrose preference was calculated as a percent of total fluid intake.

### Electrophysiology

#### *In vivo* extracellular recordings

*In vivo* extracellular recordings of the VTA were performed as described previously (Renard et al., 2017, 2018; Szkudlarek et al., 2019), on treatment groups between PD100 and PD120. Following behavioral testing, a subset of rats was anesthetized with urethane (1.4 g/kg, i.p.), and placed in a stereotaxic frame with their body temperature maintained between 36–37°C. A scalp incision was made, and a hole was drilled in the skull overlaying the VTA. Three glass microelectrodes (2 mm diameter), produced with the PE-21 Microelectrode Puller (Narishige), with an average impedance of 6–10 MΩ, filled with 2% pontamine sky blue solution were lowered into each brain region using a hydraulic micropositioner (Kopf 640). The stereotaxic coordinates of the recordings were: VTA, AP −5.1 to −5.3 mm, ML ±0.7 to ±1.0 mm from bregma, and DV −7.0 to −9.0 mm from dural surface. Recordings were taken from putative DAergic neurons in the VTA. The extracellular recordings were amplified (5000×) using MultiClamp700B amplifier (Molecular Devices), digitized at 25 kHz and recorded through a Digidata1440A acquisition system (Molecular Devices) and pClamp10 software. Wideband VTA signals were separated into two channels through the digitizer and filtered to obtain single unit recordings (band pass between 0.3 and 3 kHz), and local field potentials (LFPs; low pass of 0.3 kHz); only single unit recordings were assessed. Histologic analyses were used to determine whether putative neurons are in their respective regions and were removed from analysis otherwise, as described elsewhere (Renard et al., 2017, 2018; Szkudlarek et al., 2019). Neural recordings of the VTA were determined to be DAergic depending on several specific electrophysiological criteria described previously (Renard et al., 2017, 2018; Szkudlarek et al., 2019).

### Imaging mass spectrometry

Following behavioral experiments, on PD120, all rats not allotted to electrophysiology were given an overdose of sodium pentobarbital (240 mg/kg, euthanyl), brains rapidly removed, flash frozen with dry ice, and then stored at −80°C. A subset of these brains were used for imaging mass spectrometry, while the remainder was used for Western blotting protein analyses. Mass spectrometric analyses were conducted using an AB Sciex 5800 MALDI TOF/TOF (matrix-assisted laser desorption/ionization time-of-flight) system. MALDI IMS allows for the combination of mass spectral and spatial information generating abundance heat maps of mass-to-charge (*m/z*) values of interest. Brain tissue from PCE offspring was collected on PD21 (male: *n* = 8/treatment, female: *n* = 12/treatment) and PD120 (male: *n* = 12/treatment, female: *n* = 12/treatment). Tissue was sectioned using a cryostat tissue slicer (CM 1850, Leica Biosystems; Thermo-Fisher Scientific CryoStar NX50) at −25°C with a 14-μm thickness. NAc brain sections obtained were from AP: +2.28 mm to +2.76 mm from bregma (The Rat Brain in Stereotaxic Coordinates 6th Edition). Brain tissue was thaw-mounted onto conductive indium tin oxide (ITO)-coated glass slides (Hudson Surface Technology Inc.). Glass slides were then stored at −80°C. Before matrix sublimation, slides were placed in a desiccator for 45 min. The matrix sublimation process is reported elsewhere (Ibrahim et al., 2017; Chen, 2021). DPH was selected as the matrix as it was reported to facilitate efficient ionization of fatty acids and phospholipids present in the tissue with MALDI IMS (Ibrahim et al., 2017). Profiling data were processed using Data Explorer (AB Sciex), and imaging data were processed using MSiReader (version 0.09, FTMS Laboratory for Human Health Research, North Carolina State University). Acquisition software from Sciex was used. A 349-nm Nd:YLF “OptiBeam On-Axis” laser with a pulse rate of 400 Hz. External calibration was done at ±50 ppm in reflectron negative mode. Mass range from 80 to 450 *m/z* was used with a laser step of 70 μm.

Average mass spectra were exported at the mass-to-charge (*m/z*) values of interest in the selected regions of interest (ROIs) area under the curve (AUC) calculations were automated using a code written by the authors in MATLAB (2019a, The MathWorks). AUC data were then standardized as the ratio THC/VEH, i.e., the change in AUC induced by PCE relative to VEH. To minimize run-to-run variation in IMS signal intensities, the ratioing of THC/VEH was done between pairs of brain tissues mounted on the same ITO glass slides, keeping the thickness of DPH layer and mass spectrometric acquisition parameters consistent for each THC/VEH pair.

Lipid fragmentation can occur in MALDI to produce fatty acid fragments indistinguishable from the endogenous fatty acids on the tissue. However, the laser energy used in this work has been kept moderately low to minimize fragmentation. Thus, we are confident that the detected decreases in fatty acid signals are in fact because of decreases in free fatty acids in the tissue as opposed to fragmentation artifacts.

### Protein extraction and Western blotting

Following electrophysiological experiments, on PD120, all rats not allotted to electrophysiology, were given an overdose of sodium pentobarbital (240 mg/kg, euthanyl), brains rapidly removed, flash frozen with dry ice, and then stored at −80°C. Regions were sectioned with a brain block to obtain bilateral NAc punchouts. Samples were homogenized using a Dounce homogenizer containing protein extraction lysis buffer (NaCl, Tris pH 8.0, 1% NP-40, 10% glycerol, and 0.1% SDS) with 1:100 protease and phosphatase inhibitors included (Halt 100× inhibitor cocktail, ThermoFisher). The sample was then centrifuged at 10,000 rpm for 15 min at 4°C, to remove insoluble material. A 20-μl aliquot was removed for protein counting using a Pierce BCA Protein Assay kit. The remaining sample solution was then mixed with an equal volume of 2× laemmli loading buffer, vortexed, and then heated at 95°C for 5 min before storage at −80°C. The Western blotting procedure was performed as previously described (Renard et al., 2017, 2018; Szkudlarek et al., 2019). Either 20 or 40 μg/well of the stored protein from the control and PCE samples was loaded onto either an 8% or 10% denaturing SDS-PAGE gels. These gels were subjected to electrophoresis, using a Bio-Rad Mini Protein 2 Western blotting apparatus with Tris/glycine/SDS buffer (Bio-Rad Cube Solutions) at 125 V for 1.5 h. The protein was then transferred onto a nitrocellulose membrane (Bio-Rad) using a Mini Trans-Blot Transfer apparatus (Bio-Rad) with a Tris/glycine/ethanol solution (Bio-Rad Cube Solutions). Membranes were then blocked with either 2.5% or 5.0% nonfat dry milk in Tris=buffered saline with Tween 20 (TBS-T) for 1 h with rocking, at room temperature. The membranes were then incubated overnight in a solution of bovine serum albumin (Sigma) in TBS-T with a primary antibody of interest at 4°C with rocking. Primary antibody dilutions were as follows: α-tubulin (1:15,000, rabbit, Sigma-Aldrich; 1:10,000, mouse, Santa Cruz Biotechnology), GAD67 (1:1000; Cell Signaling Technology), dopamine D1/D2 receptor (1:750; EMD Millipore), NMDAR2A/2B receptor (1:1000; Sigma-Aldrich), PPARα/ɣ1,2 (1:750; Sigma-Aldrich), gephyrin (1:1000; Cell Signaling Technology), vGlut1/2 (1:1000; Cell Signaling Technology), p-GSKα/β and t-GSK (1:1000; Cell Signaling Technology), and synaptophysin (1:1000; Cell Signaling Technology). Following this, blots were incubated for 1 h at room temperature with appropriate secondary antibodies (Li-Cor IRDye 680RD, IRDye 800CQ-conjugated secondary antibodies, 1:15,000) in either 2.5% or 5% nonfat dry milk in TBS-T. Proteins of interest were imaged using a LI-COR Odyssey imaging system, and densitometry measurements were obtained using Image Studio digital analysis software. Relative band density was normalized to the density of each sample’s respective α tubulin.

### Statistical analyses

THC-treated rats were analyzed with separate two-way ANOVAs (sex × treatment) for each behavioral assay, electrophysiology, and for Western blot protein analyses. Follow-up analyses of significant (*p* < 0.05) main effects and interactions was accomplished using Tukey’s HSD *post hoc* test (α = 0.05). One-sample *t* tests (*p* < 0.05) with a hypothetical mean of 1.0 was conducted on all MALDI IMS ratios; 1.0 represents no difference between treatments and is treated as the values of the VEH/CT group. This method of calculation is the standard used to assess MALDI IMS results (Ibrahim et al., 2017). All analyses were performed using GraphPad Prism (version 9.0.0 for Windows 10), and exact values ± SEM are reported.

## Results

### Prenatal THC induced aberrations to anxiety but not reward-seeking behavior, anhedonia, or locomotory activity in adulthood

**Figure 1.**
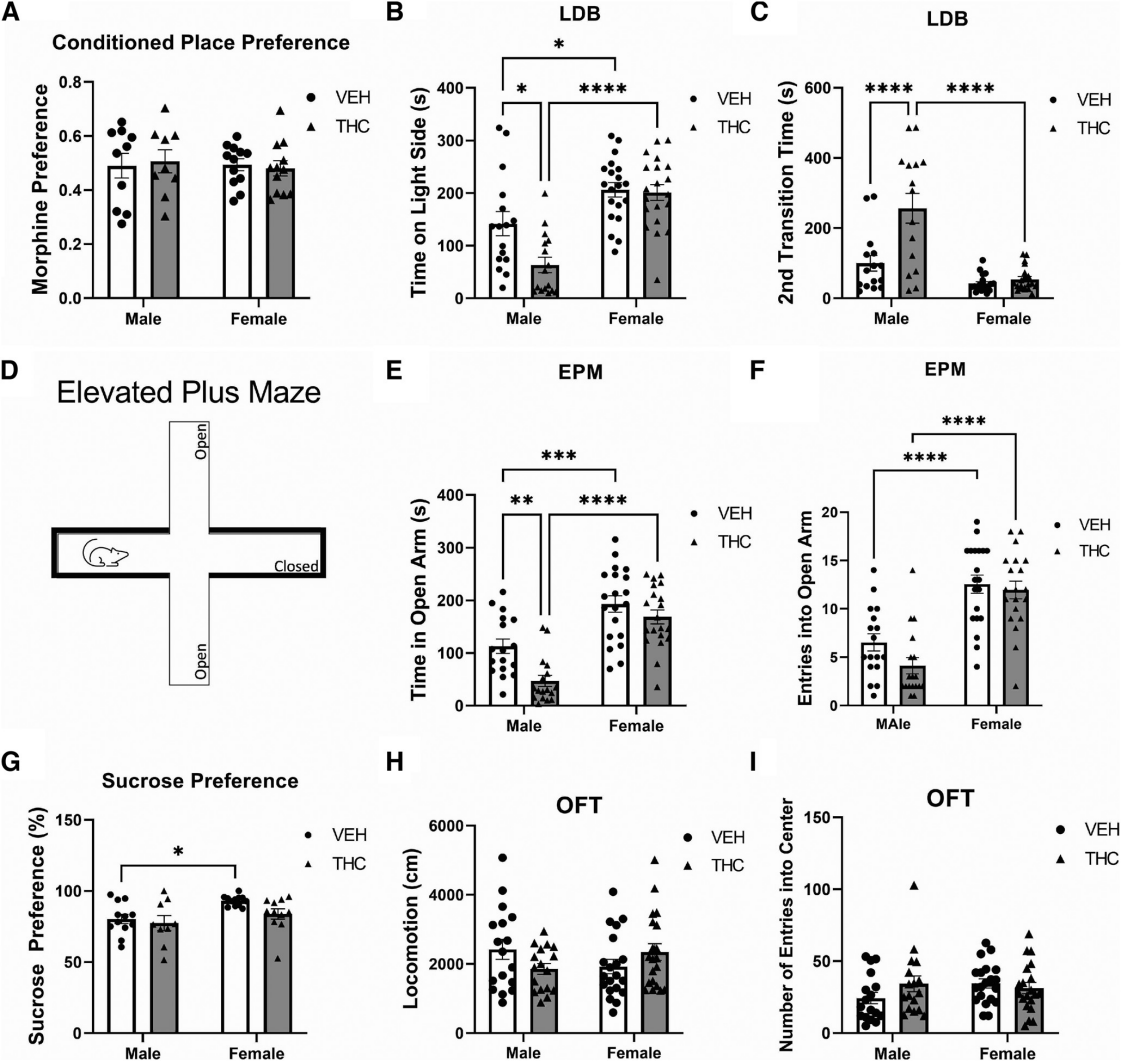
Prenatal THC induced sex-specific aberrations to anxiety. ***A***, Assessing reward-seeking behavior with the subthreshold morphine conditioned place preference test, prenatal THC did not induce any alteration in either male or female progeny (*n* = 12/treatment/sex). ***B***, ***C***, In the light/dark box (LDB), prenatal THC (male: *n* = 17/treatment; female: *n* = 20/treatment) induced significant sex-specific alterations to anxiety, with (***B***) only the exposed males spending significantly less time in the light side and (***C***) took longer to re-enter the light side. ***D***, Schematic representation of the elevated plus maze (EPM). ***E***, Prenatal THC induced significant sex-specific alterations to anxiety, with only the exposed males spending significantly less time in the open arms. ***F***, PCE did not alter either male or female open arm entry. ***G***, Prenatal THC induced significant sex-specific alterations to anhedonia, with only the female progeny exhibiting an anhedonic phenotype (*n* = 12/treatment/sex)***. H***, ***I***, Prenatal THC did not affect (***H***) male or female locomotion, nor did it effect (***I***), thigmotaxis; the total number of entries into center within the first 5 min. Comparisons were made with two-way ANOVA followed by Tukey’s HSD *post hoc* test, ****p < 0.0001, ***p < 0.001, **p < 0.01, *p < 0.05, two-tailed. *Figure Contributions*: Mohammed H. Sarikahya, Marta De Felice, and Tony Jung performed the experiments. Mohammed H. Sarikahya and Marta De Felice analyzed the experiments.

We first examined whether reward-seeking behavior was altered by PCE, given that the NAc is critically involved in reward salience processing, using a conditioned place preference (CPP) procedure and a subreward threshold conditioning dose of morphine (0.05 mg/kg, i.p.). Two-way ANOVA revealed no effects of treatment or sex on CPP test scores, with groups spending similar amounts of time in saline or morphine-paired test environments (Interaction, *F*_(1,39)_ = 0.1894, *p* = 0.6658; Sex, *F*_(1,39)_ = 0.1033, *p* = 0.7496; Treatment, *F*_(1,40)_ = 0.002494, *p* = 0.9604; Fig. 1*A*). Thus, PCE does not appear to increase sensitivity to the conditioned rewarding effects of this specific dosage of morphine in male or female offspring, as measured in the CPP paradigm.

We next assessed whether PCE induced anxiogenic-like behaviors. Two-way ANOVA revealed that, in the light dark box (LDB), the total time spent in light chamber (Fig. 1*B*) and the latency to first re-transition back from dark to light chamber (Fig. 1*C*), was effected by sex, treatment, and interaction between factors (time on light side: Interaction, *F*_(1,68)_ = 4.878, *p* = 0.0306; Sex, *F*_(1,68)_ = 37.10, *p* < 0.0001; Treatment, *F*_(1,68)_ = 6.362, *p* = 0.0140; second transition latency: Interaction, *F*_(1,68)_ = 11.36, *p* = 0.0013; Sex, *F*_(1,68)_ = 37.01, *p* < 0.0001; Treatment, *F*_(1,68)_ = 15.57, *p* = 0.0002). *Post hoc* analysis revealed that male (*n* = 17/treatment), but not female (*n* = 20/treatment), PCE progeny spent significantly more time in the dark versus light chamber (males: *p* = 0.0118; females: *p* = 0.9954; Fig. 1*B*), as well as taking significantly longer to re-transition from dark to light chamber (males: *p* < 0.0001; females: *p* = 0.9714; Fig. 1*C*).

Two-way ANOVA further exhibited that the total time spent in the EPM’s open arm (Fig. 1*D*,*E*), was effected by sex and treatment, and entries into the open arm were effected only by sex (Fig. 1*F*; time in open arm: Interaction, *F*_(1,70)_ = 2.313, *p* = 0.1328; Sex, *F*_(1,70)_ = 54.51, *p* < 0.0001; Treatment, *F*_(1,70)_ = 10.98, *p* = 0.0015; Open Arm Entries: Interaction, *F*_(1,70)_ = 1.023, *p* = 0.3151; Sex, *F*_(1,70)_ = 59.46, *p* < 0.0001; Treatment, *F*_(1,70)_ = 2.820, *p* = 0.0975). *Post hoc* analysis exhibited that only male PCE progeny spent significantly less time in open arms versus VEH controls (males: *p* = 0.0084; females: *p* = 0.5516; Fig. 1*E*), with no effect observed with open arm entry (males: *p* = 0.2626; females: *p* = 0.9614; Fig. 1*F*)

Next, we assessed anhedonic-like phenotypes with the sucrose preference test (*n* = 12/sex/treatment; Fig. 1*G*). Two-way ANOVA revealed only sex to have an effect on sucrose preference (Interaction, *F*_(1,40)_ = 1.671, *p* = 0.3560; Sex, *F*_(1,40)_ = 15.56, *p* = 0.0078; Treatment, *F*_(1,40)_ = 6.075, *p* = 0.0826), with *post hoc* analyses exhibiting no significant sucrose preference in either males or females (males: *p* = 0.9366; females: *p* = 0.2148; Fig. 1*G*).

Lastly, we assessed motility and thigmotaxis. Two-way ANOVA revealed that in the open field test (OFT; *n* = 8/sex/treatment), neither motility (Fig. 1*H*) nor entries into center (i.e., thigmotaxis; Fig. 1*I*) were effected by any factors (motility: Interaction, *F*_(1,70)_ = 3.173, *p* = 0.0857; Sex, *F*_(1,70)_ = 0.2045, *p* = 0.6546; Treatment, *F*_(1,70)_ = 0.07,823, *p* = 0.7818; thigmotaxis: Interaction, *F*_(1,70)_ = 0.8945, *p* = 0.3524; Sex, *F*_(1,70)_ = 0.008868, *p* = 0.9256; Treatment, *F*_(1,70)_ = 1.768, *p* = 0.1943); *post hoc* analyses could not be conducted. Female estrous cycles had no effect on any behavioral outcome (data not shown).

### Prenatal THC exposure induces long-term sex-specific alterations in VTA neuronal activity

**Figure 2.**
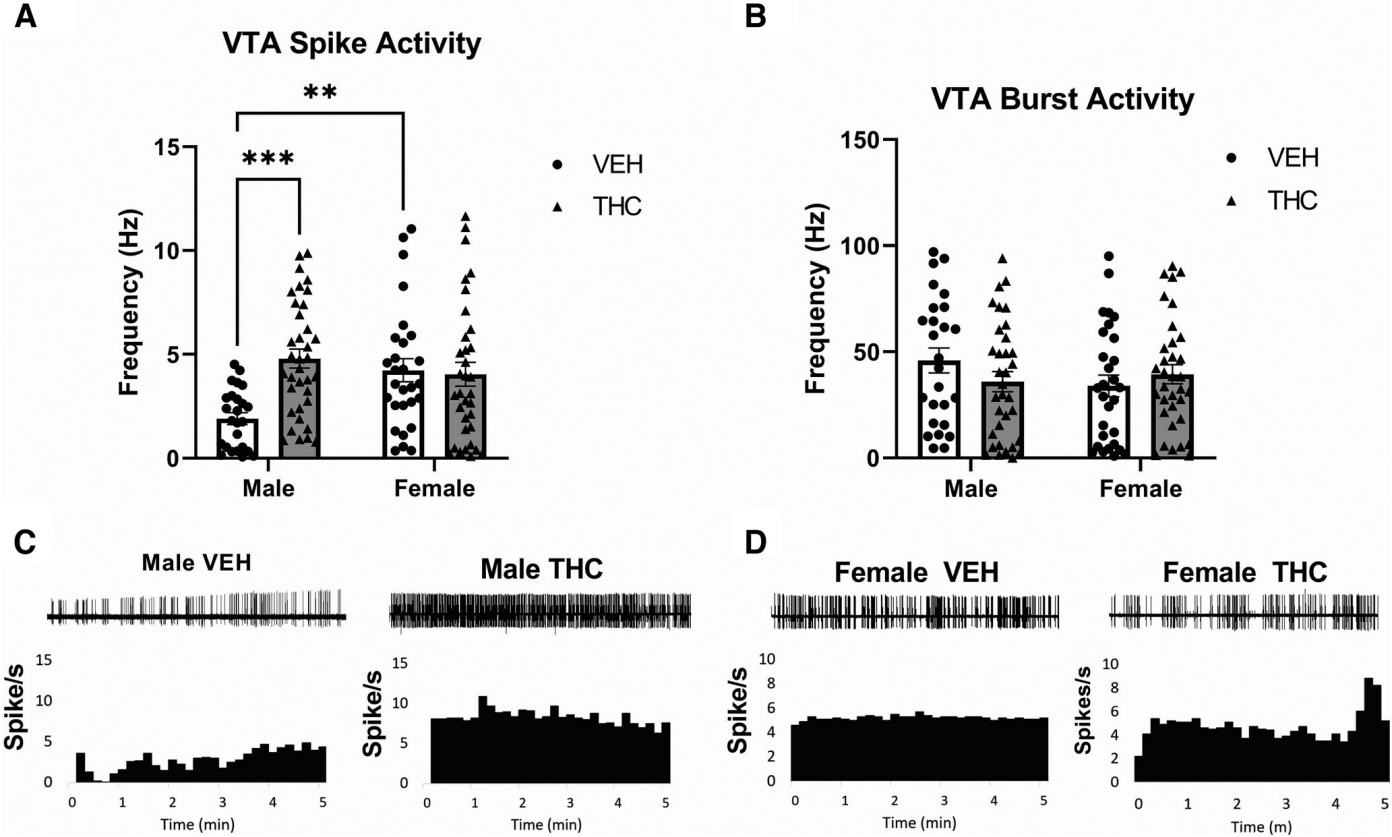
Prenatal THC induces sex-specific alterations to the Ventral Tegmental Area. ***A***, Prenatal THC induces significantly higher spontaneous dopamine firing frequency in the male PCE progeny’s VTA (vehicle *n* = 28 from 5 rats, THC *n* = 36 from 6 rats), but not the female progeny (vehicle *n* = 26 from 5 rats, THC *n* = 35 from 5 rats). ***B***, VTA bursting activity was not affected in either male or female progeny. ***C***, ***D***, Traces and rate histograms of representative VTA dopaminergic neurons from (***C***) male and (***D***) female progeny. Comparisons were made with two-way ANOVA followed by Tukey’s HSD *post hoc* test, ***p < 0.001, **p < 0.01. *Figure Contributions*: Mohammed H. Sarikahya, Marta De Felice, and Marieka DeVuono performed the experiments. Mohammed H. Sarikahya and Marta De Felice analyzed the experiments.

The dysregulation of subcortical DAergic activity states, namely enhanced excitability in the VTA, are observable in other models of PCE and adolescent THC exposure models (Renard et al., 2018; Frau et al., 2019; Sagheddu et al., 2021; Traccis et al., 2021); thus, we examined VTA DAergic neuronal activity in order replicate these previous findings and to associate them with behavioral outcomes. Two-way ANOVA revealed that VTA DA neuron spiking activity (Fig. 2*A*) was effected by sex and treatment, but VTA DA neuron bursting rate (Fig. 2*B*) was not effected by any interaction (VTA firing frequency: Interaction, *F*_(1,119)_ = 9.349, *p* = 0.0028; Sex, *F*_(1,119)_ = 2.414, *p* = 0.1229; Treatment, *F*_(1,119)_ = 7.123, *p* = 0.0087; Bursting Rate: *F*_(1,119)_ = 2.346, *p* = 0.1282; Sex, *F*_(1,119)_ = 0.7159 *p* = 0.3992; Treatment, *F*_(1,119)_ = 0.1985, *p* = 0.6568). *Post hoc* Tukey’s HSD further revealed that only male PCE progeny exhibit significant baseline firing activity (male: *p* = 0.0001; female: *p* = 0.7818**;** Fig. 2*A*). *Post hoc* analyses could not be conducted on VTA bursting rate. Female estrous cycles had no effect on neuronal outcomes (data not shown).

### Prenatal THC exposure induces alterations in brain PUFA concentrations during prepubescence that persist into adulthood

**Figure 3.**
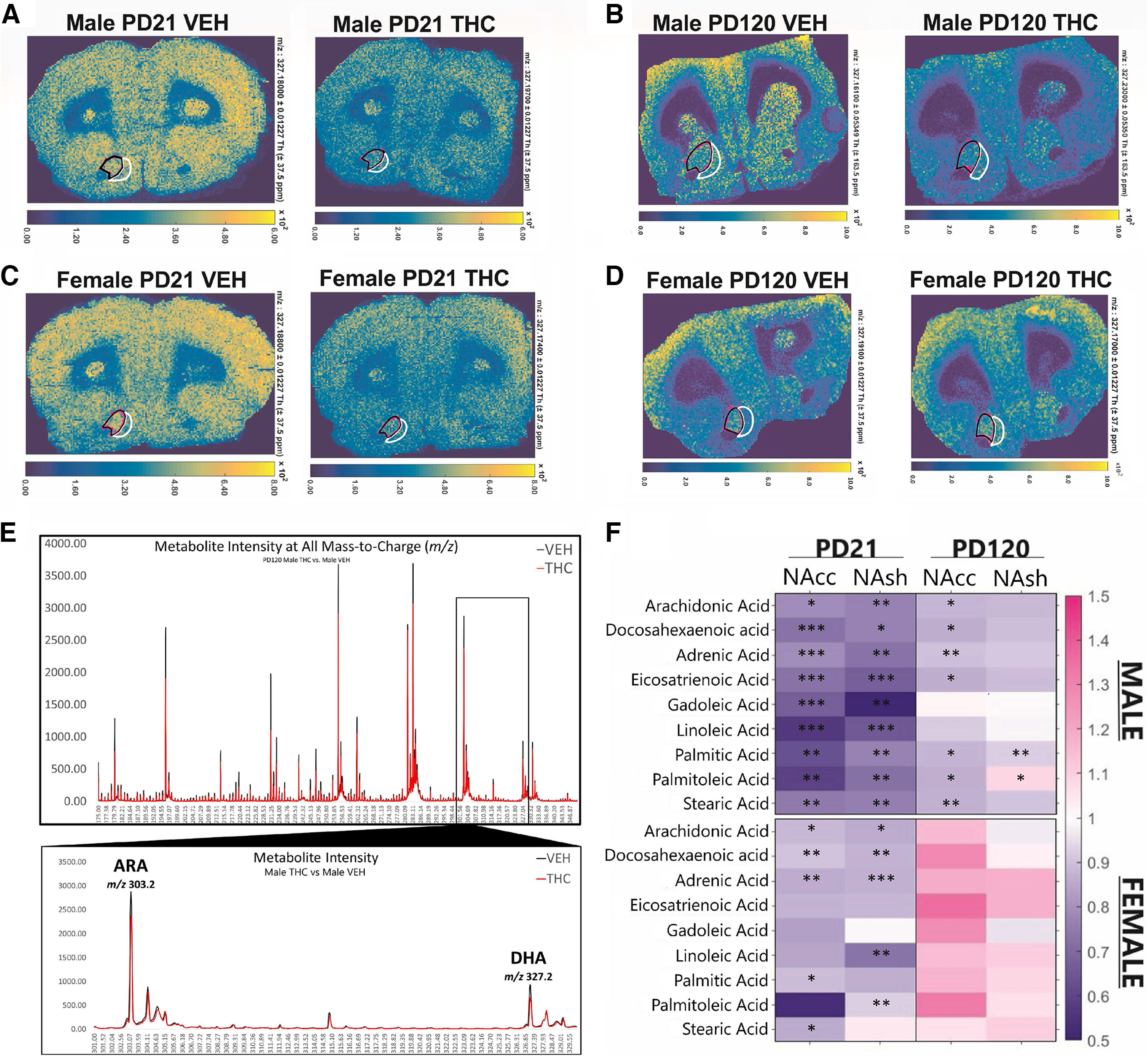
Prenatal THC induces significant alterations to fatty acid concentrations in the nucleus accumbens. ***A–D***, Representative MALDI IMS image at (***A***) PD21 male VEH and THC exposed progeny, (***B***) PD120 male VEH and THC exposed progeny, (***C***) PD21 female VEH and THC progeny, and (***D***) PD120 female VEH and THC progeny, exhibiting the relative intensities for the mass target for DHA (*m/z* 327.23) in the nucleus accumbens core (NAcc) and shell (NAsh). The NAcc (black) and NAsh (white) for one hemisphere are highlighted in each IMS image. ***E***, Intensity plot of representative PD21 male VEH and THC progeny. All experimental masses identified are within reasonable and expected variance from theoretical *m/z* values. ***F***, Data presented is the mean area under the curve (AUC) of relevant peak intensities comparing the THC to VEH (THC/VEH) relative values; a ratio of 1.0 suggests no difference between the treatments; the standard method of analyzing MALDI IMS data (Ibrahim et al., 2017). Fatty acids were detected as the deprotonated acids, [M-H]**^–^**; DHA *m/z* 327.23, ARA *m/z* 303.23, adrenic acid *m/z* 331.23, eicosatrienoic acid *m/z* 305.20, gadoleic acid *m/z* 309.25, linoleic acid *m/z* 279.20, palmitic acid *m/z* 255.21, palmitoleic acid *m/z* 253.19, and stearic acid *m/z* 283.23. The NAcc and NAsh of PD21 male (*n* = 8/treatment) and female (*n* = 10/treatment), and PD120 male (*n* = 12/treatment) and female (*n* = 12/treatment) were assessed. The male and female progeny exhibit distinct and significant reductions across several fatty acids in a region-dependent manner. See Extended Data Figures 3-1, 3-2, and 3-3 for representative MALDI IMS images for each assessed fatty acid. Comparisons were conducted with one-sample *t* tests with a hypothetical mean of 1.0, ***p < 0.001, **p < 0.01, *p < 0.05, two-tailed. *Figure Contributions*: Sammy Cousineau performed the MALDI IMS imaging experiments. Mohammed H. Sarikahya and Sammy Cousineau analyzed the experiments.

We next assessed a key component of the lipidome, the PUFAs, DHA and ARA, considering their role in DAergic neurotransmission, synaptic integrity and function, and are functionally intertwined with the ECb system (Kidd, 1996; Mozzi et al., 2003; Shevchenko and Simons, 2010; Wood et al., 2014; Zhuo et al., 2020), which suggests potential vulnerability to the effects of PCE. In fact, previous low dose PCE (2 mg/kg) studies suggest alterations to synaptic integrity (Frau et al., 2019; Scheyer et al., 2019; Sagheddu et al., 2021; Traccis et al., 2021). Experimental PUFA data, with MALDI IMS, was tracked using theoretical masses, as determined by LIPID MAPS (Wellcome Trust) and were confirmed as experimental mass targets by other well-validated MS methods (Ibrahim et al., 2017). Fatty acids were detected as the deprotonated acids, [M-H]**^–^**; DHA *m/z* 327.23 (Fig. 3*A–D*) and ARA *m/z* 303.23 (Extended Data Fig. 3-1*A*), adrenic acid (AA) *m/z* 331.23 (Extended Data Fig. 3-1*B*), eicosatrienoic acid *m/z* 305.20 (Extended Data Fig. 3-1*C*), gadoleic Acid *m/z* 309.25 (Extended Data Fig. 3-2*A*), linoleic acid *m/z* 279.20 (Extended Data Fig. 3-2*B*), palmitic acid *m/z* 255.21 (Extended Data Fig. 3-2*C*), palmitoleic acid *m/z* 253.19 (Extended Data Fig. 3-3*A*), and stearic acid *m/z* 283.23 (Extended Data Fig. 3-3*B*; Ibrahim et al., 2017). Substantial sex-specific alterations across all PUFAs and monounsaturated fatty acids (MUFA) was observed in the NAc core (NAcc) and shell (NAsh) at PD21 and PD120, with the female progeny exhibiting a recovery of their observed PUFA/MUFA deficits, while the PCE-induced alterations endured in the male progeny (Fig. 3*F*; see Table 1).

**Table 1:** Male and female NAcc and NAsh MALDI IMS fatty acid analyses at PD21 and PD120

Fatty acid	Mass-to-charge ratio (*m/z*)	Mean AUC THC/VEH	*t* statistic, df	Exact *p* value
Male NAcc PD21	*** ***	*** ***	** **
Arachidonic acid (ARA)	*m/z* 303.23	0.7916	*t* = 3.137, df = 7	0.0139*
Docosahexaenoic acid (DHA)	*m/z* 327.23	0.7302	*t* = 6.574, df = 7	0.0003***
Adrenic acid	*m/z* 331.23	0.792	*t* = 5.384, df = 7	0.0007***
Eicosatrienoic acid	*m/z* 305.20	0.73	*t* = 5.643, df = 7	0.0005***
Gadoleic acid	*m/z* 309.25	0.671	*t* = 5.456, df = 7	0.0006***
Linoleic acid	*m/z* 279.20	0.568	*t* = 5.672, df = 7	0.0005***
Palmitic acid	*m/z* 255.21	0.632	*t* = 3.669, df = 7	0.0063**
Palmitoleic acid	*m/z* 253.19	0.59	*t* = 3.538, df = 7	0.0076**
Stearic acid	*m/z* 283.23	0.748	*t* = 3.516, df = 7	0.0079**
Male NAcc PD120	*** ***	*** ***	** **
Arachidonic acid (ARA)	*m/z* 303.23	0.8782	*t* = 2.908, df = 11	0.0131*
Docosahexaenoic acid (DHA)	*m/z* 327.23	0.8913	*t* = 2.592, df = 11	0.0269*
Adrenic acid	*m/z* 331.23	0.902	*t* = 3.264, df = 11	0.0068**
Eicosatrienoic acid	*m/z* 305.20	0.862	*t* = 2.727, df = 11	0.0184*
Gadoleic acid	*m/z* 309.25	1.012	*t* = 0.1017, df = 11	0.9207
Linoleic acid	*m/z* 279.20	0.921	*t* = 0.9614, df = 11	0.3553
Palmitic acid	*m/z* 255.21	0.878	*t* = 2.221, df = 11	0.0463*
Palmitoleic acid	*m/z* 253.19	0.867	*t* = 2.981, df = 11	0.0115*
Stearic acid	*m/z* 283.23	0.868	*t* = 3.282, df = 11	0.0066**
Male NAsh PD21	*** ***	*** ***	** **
Arachidonic acid (ARA)	*m/z* 303.23	0.7693	*t* = 5.503, df = 7	0.0009***
Docosahexaenoic acid (DHA)	*m/z* 327.23	0.7676	*t* = 3.819, df = 7	0.0066**
Adrenic acid	*m/z* 331.23	0.73	*t* = 4.893, df = 7	0.0018**
Eicosatrienoic acid	*m/z* 305.20	0.672	*t* = 7.089, df = 7	0.0002***
Gadoleic acid	*m/z* 309.25	0.494	*t* = 5.256, df = 7	0.0012**
Linoleic acid	*m/z* 279.20	0.649	*t* = 6.087, df = 7	0.0005***
Palmitic acid	*m/z* 255.21	0.769	*t* = 4.881, df = 7	0.0018**
Palmitoleic acid	*m/z* 253.19	0.722	*t* = 3.826, df = 7	0.0065**
Stearic acid	*m/z* 283.23	0.76	*t* = 5.610, df = 7	0.0008**
Male NAsh PD120	*** ***	*** ***	** **
Arachidonic acid (ARA)	*m/z* 303.23	0.8931	*t* = 2.908; df = 11	0.0131*
Docosahexaenoic acid (DHA)	*m/z* 327.23	0.9117	*t* = 1.982; df = 11	0.0756
Adrenic acid	*m/z* 331.23	0.912	*t* = 0.1012; df = 11	0.0526
Eicosatrienoic acid	*m/z* 305.20	0.893	*t* = 2.150; df = 11	0.9196
Gadoleic acid	*m/z* 309.25	0.994	*t* = 0.1031; df = 11	0.0782
Linoleic acid	*m/z* 279.20	0.988	*t* = 0.8355; df = 11	0.5898
Palmitic acid	*m/z* 255.21	0.922	*t* = 1.897; df = 11	0.0076**
Palmitoleic acid	*m/z* 253.19	1.084	*t* = 1.497; df = 11	0.0280*
Stearic acid	*m/z* 283.23	0.905	*t* = 2.065; df = 11	0.0526
Female NAcc PD21	*** ***	*** ***	** **
Arachidonic acid (ARA)	*m/z* 303.23	0.8577	*t* = 2.685, df = 9	0.025*
Docosahexaenoic acid (DHA)	*m/z* 327.23	0.8738	*t* = 3.592, df = 9	0.0058**
Adrenic acid	*m/z* 331.23	0.858	*t* = 3.392, df = 9	0.0095**
Eicosatrienoic acid	*m/z* 305.20	0.874	*t* = 1.746, df = 9	0.1243
Gadoleic acid	*m/z* 309.25	0.833	*t* = 1.975, df = 9	0.0838
Linoleic acid	*m/z* 279.20	0.845	*t* = 1.421, df = 9	0.1931
Palmitic acid	*m/z* 255.21	0.893	*t* = 3.120, df = 9	0.0142*
Palmitoleic acid	*m/z* 253.19	0.524	*t* = 1.871, df = 9	0.0983
Stearic acid	*m/z* 283.23	0.857	*t* = 2.872, df = 9	0.0208*
Female NAcc PD120	*** ***	*** ***	** **
Arachidonic acid (ARA)	*m/z* 303.23	1.153	*t* = 2.152, df = 11	0.0569
Docosahexaenoic acid (DHA)	*m/z* 327.23	1.282	*t* = 1.405, df = 11	0.1805
Adrenic acid	*m/z* 331.23	1.195	*t* = 1.274, df = 11	0.2567
Eicosatrienoic acid	*m/z* 305.20	1.352	*t* = 1.513, df = 11	0.1805
Gadoleic acid	*m/z* 309.25	1.272	*t* = 0.8198, df = 11	0.222
Linoleic acid	*m/z* 279.20	1.147	*t* = 1.274, df = 11	0.4252
Palmitic acid	*m/z* 255.21	1.172	*t* = 0.6142, df = 11	0.222
Palmitoleic acid	*m/z* 253.19	1.321	*t* = 1.421, df = 11	0.1022
Stearic acid	*m/z* 283.23	1.056	*t* = 0.9534, df = 11	0.5483
Female NAsh PD21	*** ***	*** ***	** **
Arachidonic acid (ARA)	*m/z* 303.23	0.9025	*t* = 2.820, df = 9	0.0201*
Docosahexaenoic acid (DHA)	*m/z* 327.23	0.871	*t* = 3.370, df = 9	0.0083**
Adrenic acid	*m/z* 331.23	0.871	*t* = 5.995, df = 9	0.0002***
Eicosatrienoic acid	*m/z* 305.20	0.88	*t* = 2.111, df = 9	0.061
Gadoleic acid	*m/z* 309.25	0.989	*t* = 0.1282, df = 9	0.9005
Linoleic acid	*m/z* 279.20	0.734	*t* = 3.785, df = 9	0.0036**
Palmitic acid	*m/z* 255.21	0.867	*t* = 1.899, df = 9	0.0868
Palmitoleic acid	*m/z* 253.19	0.923	*t* = 3.332, df = 9	0.0088**
Stearic acid	*m/z* 283.23	1.041	*t* = 0.8969, df = 9	0.3931
Female NAsh PD120	*** ***	*** ***	** **
Arachidonic acid (ARA)	*m/z* 303.23	0.8884	*t* = 1.197, df = 11	0.2566
Docosahexaenoic acid (DHA)	*m/z* 327.23	0.9297	*t* = 0.6804, df = 11	0.5103
Adrenic acid	*m/z* 331.23	1.18	*t* = 0.1237, df = 11	0.904
Eicosatrienoic acid	*m/z* 305.20	1.177	*t* = 0.1136, df = 11	0.9116
Gadoleic acid	*m/z* 309.25	0.957	*t* = 1.981, df = 11	0.0757
Linoleic acid	*m/z* 279.20	1.106	*t* = 1.197, df = 11	0.2566
Palmitic acid	*m/z* 255.21	1.084	*t* = 0.3331, df = 11	0.7453
Palmitoleic acid	*m/z* 253.19	1.061	*t* = 0.9200, df = 11	0.3773
Stearic acid	*m/z* 283.23	1.098	*t* = 1.041, df = 11	0.3224

Mass-to-charge (*m/z*) values obtained in Nucleus Accumbens Core (NAcc) and Nucleus Accumbens Shell (NAsh) are based on previously described mass targets (Ibrahim et al., 2017).

Area under the curve (AUC) ratio comparisons were conducted with one-sample *t* tests, with a hypothetical mean of 1.0, where 1.0 suggests no difference between the VEH and THC offspring; ****p < 0.0001, ***p < 0.001, **p < 0.01, *p < 0.05, two-tailed.

10.1523/ENEURO.0253-22.2022.f3-1 Extended Data Figure 3-1 ***A***, Representative MALDI IMS image for arachidonic acid for PD120 male VEH versus THC progeny. ***B***, Representative MALDI IMS images for adrenic acid for PD120 male VEH versus THC progeny. ***C***, Representative MALDI IMS images for eicosatrienoic acid for PD120 male VEH versus THC progeny. Download Figure 3-1, TIF file.

10.1523/ENEURO.0253-22.2022.f3-2 Extended Data Figure 3-2 ***A***, Representative MALDI IMS image for gadoleic acid for PD120 male VEH versus THC progeny. ***B***, Representative MALDI IMS images for linoleic acid for PD120 male VEH versus THC progeny. ***C***, Representative MALDI IMS images for palmitic acid for PD120 male VEH versus THC progeny. Download Figure 3-2, TIF file.

10.1523/ENEURO.0253-22.2022.f3-3 Extended Data Figure 3-3 ***A***, Representative MALDI IMS image for palmitoleic acid for PD120 male VEH versus THC progeny. ***B***, Representative MALDI IMS images for stearic acid for PD120 male VEH versus THC progeny. Download Figure 3-3, TIF file.

### Prenatal THC exposure induces sex-specific alterations in neuropsychiatric molecular biomarkers in the NAc that persist into adulthood

We next examined expression levels of several molecular signaling pathways known to be disrupted following neurodevelopmental adolescent THC exposure and dysregulated in neuropsychiatric disorders (Ohtsuki et al., 2001; Madras, 2013; Urs et al., 2017; Renard et al., 2018; Szkudlarek et al., 2019; De Felice and Laviolette, 2021). Given that our VTA data revealed significant disruptions in DAergic activity states, we first characterized intra-NAc DA D1/D2R expression patterns. At PD21, two-way ANOVA revealed that only an interaction between factors, but not sex or treatment, effect D2R expression (Interaction, *F*_(1,28)_ = 7.538, *p* = 0.0108; Sex, *F*_(1,28)_ = 0.4229, *p* = 0.5212; Treatment, *F*_(1,28)_ = 3.089, *p* = 0.0906); with *post hoc* analyses revealing a significant decrease in the male (*p* = 0.0233), but not female (*p* = 0.8870), progeny. At PD120, two-way ANOVA revealed that the interaction between factors, sex, and treatment effect D2R expression (Interaction, *F*_(1,28)_ = 10.20, *p* = 0.0037; Sex, *F*_(1,28)_ = 11.19, *p* = 0.0025; Treatment, *F*_(1,28)_ = 7.642, *p* = 0.0103); with *post hoc* analyses revealing a significant decrease in the male (*p* = 0.0014), but not female (*p* = 0.9900), progeny. Thus, PCE induces life-long dopamine receptor expression alterations.

**Figure 4.**
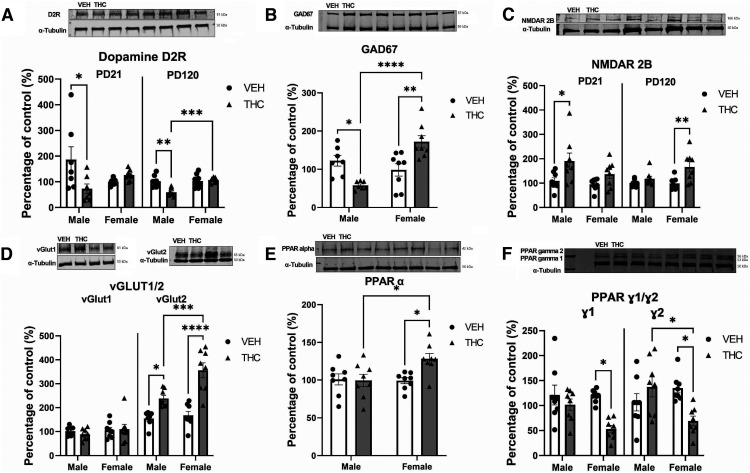
Prenatal THC induces significant alterations in molecular biomarkers in the NAc. Blots are cropped to fit both protein of interest and α-Tubulin for ***A***, ***C***, ***D*** (vGlut1 only), and ***E***. All representative Western blottings exhibit alternating VEH, THC. ***A***. Representative Western blotting of dopamine D2R. Densitometry analysis revealed that, at postnatal day (PD) 21 and PD120, only the male PCE rats (*n* = 8/treatment) have significant decreases in D2R expression relative VEH controls, while female progeny (*n* = 8/treatment) exhibited no difference. ***B***, Representative Western blotting of GAD67 at PD120. The male PCE progeny at PD120 exhibits a significant decrease, while the female progeny exhibits a significant increase in GAD67 expression relative to vehicle controls. ***C***, Representative Western blotting of NMDAR2B. At PD21, male progeny exhibits a significant increase, but at PD120, only the female PCE progeny exhibits significant increases in NMDAR2B expression. ***D***, Representative Western blottings of vGlut1 and vGlut2 at PD120. The male and female PCE progeny exhibits no significant deficits in vGlut1, but both exhibit significant increases in vGlut2. ***E***, Representative Western blotting of PPARα at PD120. The female PCE progeny exhibits a significant increase in PPARα expression, while the males exhibit no difference in expression. ***F***, Representative Western blotting of PPAR ɣ1 and ɣ2 at PD120. The female PCE progeny exhibits a significant decrease in PPAR ɣ1 and ɣ2 expression, while the males exhibit no difference in expression. Comparisons were made with two-way ANOVA followed by Tukey’s HSD *post hoc* test, ****p < 0.0001, ***p < 0.001, **p < 0.01, *p < 0.05. *Figure Contributions*: Mohammed H. Sarikahya, Karen KW Wong, Mar Rodríguez-Ruiz, Tsun Hay Jason Ng, Dana Gummerson, and Emma Proud performed the experiments. Mohammed H. Sarikahya and Karen KW Wong analyzed the experiments.

Abnormal GAD67 expression, which is involved with GABA metabolism, is associated with neuropsychiatric-like phenotypes, and is reduced following adolescent THC exposure (Renard et al., 2018; Szkudlarek et al., 2019). Two-way ANOVA suggests that both an interaction between factors and sex, effect GAD67 expression (Interaction, *F*_(1,28)_ = 24.76, *p* < 0.0001; Sex, *F*_(1,28)_ = 10.57, *p* = 0.0032; Treatment, *F*_(1,28)_ = 0.1126, *p* = 0.7399); with *post hoc* analyses revealing a significant reduction in the male progeny and significant increase in the female progeny (*p* = 0.0033), and not the male progeny (*p* = 0.0187; Fig. 4*B*). Thus, PCE differentially effects GABA metabolism in male and female progeny. No effect was observed in GAD67 expression at PD21 in either male or females (*p* > 0.05; data not shown).

Next, we assessed NMDAR2B, a glutamate receptor, to characterize GLUT/GABA interactions. At PD21, two-way ANOVA suggests that only the PCE treatment had an effect on NMDAR2B expression (Interaction, *F*_(1,28)_ = 0.8910, *p* = 0.1099; Sex, *F*_(1,28)_ = 2.725, *p* = 0.1099; Treatment, *F*_(1,28)_ = 9.017, *p* = 0.0056); with *post hoc* analyses suggesting that male (*p* = 0.0437), but not female (*p* = 0.4766), progeny have a significant increase in NMDAR2B expression. At PD120, two-way ANOVA again suggests that only the PCE treatment has an effect on NMDAR2B expression (Interaction, *F*_(1,28)_ = 3.552, *p* = 0.0703; Sex, *F*_(1,28)_ = 3.175, *p* = 0.0860; Treatment, *F*_(1,28)_ = 9.991, *p* = 0.0039); with *post hoc* analyses revealing a significant increase in NMDAR2B expression in the female (*p* = 0.0082), but not male (*p* = 0.7953), progeny. PCE induced a significant increase in the expression of NMDAR2B in female offspring relative to VEH controls (MWU = 6; *p* = 0.0093; Fig. 4*B*) with no effect observed in males (MWU = 15; *p* = 0.0830; Fig. 4*C*).

We next explored the effect of PCE on vGlut1, vGlut2, gephyrin, GSK, and synaptophysin, all of which are critical for synaptic function (Hajjar et al., 2013; Choii and Ko, 2015; Martineau et al., 2017). Two-way ANOVA suggests that both an interaction between factors, sex, and treatment effect vGlut1 and vGlut2 expression (Interaction, *F*_(3,55)_ = 15.13, *p* < 0.0001; Sex, *F*_(3,55)_ = 44.09, *p* < 0.0001; Treatment, *F*_(3,55)_ = 30.52, *p* < 0.0001); with *post hoc* analyses suggesting a significant increase in vGlut2 in both male (*p* = 0.0242) and female progeny (*p* < 0.0001; Fig. 4*D*). No effect was observed in vGlut1, vGlut2 at PD21 in either male or females (*p* > 0.05; data not shown). Lastly, PCE did not result with a sex-specific change in gephyrin, or any change in GSKα/β or synaptophysin expression levels at PD21 or PD120 in either males or females (*p* > 0.05; data not shown).

PPARs are a family of nuclear receptors critical to lipid metabolism and neuroinflammation that functionally interacts with the ECb system, DHA, ARA, and their metabolites (Watson et al., 2019; Majou, 2021). Two-way ANOVA revealed that only an interaction between factors effected PPARα expression (Interaction, *F*_(1,28)_ = 4.981, *p* = 0.0338; Sex, *F*_(1,28)_ = 3.933, *p* = 0.0572; Treatment, *F*_(1,28)_ = 4.099, *p* = 0.0525), with *post hoc* analyses suggesting a significant increase in the female (*p* = 0.0265), but not the male (*p* = 0.9988), progeny (Fig. 4*E*). Two-way ANOVA revealed that both interaction between factor and treatment effect PPARɣ1/ɣ2 expression (Interaction, *F*_(3,56)_ = 5.814, *p* = 0.0016; Sex, *F*_(3,56)_ = 2.543, *p* = 0.0654; Treatment, *F*_(3,56)_ = 9.919, *p* = 0.0026), with *post hoc* analyses suggesting that in there are significant decreases in the female progeny in both PPARɣ1 (*p* = 0.0217) and ɣ2 (*p* = 0.0232; Fig. 4*F*). In contrast, males did not exhibit any significant change in either PPARɣ isoform, including ɣ1 (*p* = 0.9715) and ɣ2 (*p* = 0.7460; Fig. 4*F*). No effect was observed in PPARα or ɣ1/2 expression at PD21 in either male or females (*p* > 0.05; data not shown).

## Discussion

The use of cannabinoid products during pregnancy for nausea, anxiety and other pregnancy-related issues is rising, particularly in jurisdictions with access to legal cannabis products. These trends are concerning, particularly because of the paucity of long-term clinical studies exploring the potential neurodevelopmental effects of PCE on offspring (Grant et al., 2018) and considering that relative concentrations of THC in cannabis products are dramatically increasing (Volkow et al., 2019). The present study demonstrates that moderate, translationally valid levels of prenatal THC exposure (Grant et al., 2018; Volkow et al., 2019; Gillies et al., 2020; Natale et al., 2020; K Lee et al., 2021) induces long-lasting, sex-specific neuropsychiatric phenotypes in both the male and female mesocorticolimbic systems. Indeed, the THC exposure protocol used in the current study represents a physiologically translational concentration such that, unlike higher dose ranges (4–5 mg/kg or higher), it does not lead to fetal demise, altered litter sizes, offspring survival rates, or maternal care parameters (Gillies et al., 2020; Natale et al., 2020; K Lee et al., 2021), alterations which, regardless of THC exposure, can alter offspring behaviors (PR Lee et al., 2007; Murgatroyd et al., 2015). Indeed, studies using higher THC doses (e.g., 5 mg/kg) have reported only sociability deficits, with no effects on anxiety, incongruent with the present data (Bara et al., 2018). However, given that alterations to maternal care are known to impact offspring social behaviors, higher dose THC exposures may be confounded with that of maternal behavioral changes and other related alterations in prenatal life experience (Hill et al., 2010; Brancato and Cannizzaro, 2018). The present THC exposure protocol thus represents a more ecologically valid translational cannabis exposure range, insofar as modeling typical human maternal cannabis consumption (Gillies et al., 2020; Natale et al., 2020; K Lee et al., 2021). Notably, the present data shows that a moderate THC dose is sufficient to induce long-term anxiety-like deficits selectively in males (Fig. 1) and corresponds to neuronal VTA DAergic hyperactivity (Fig. 2), consistent with previous studies (Frau et al., 2019; Traccis et al., 2021). More importantly, the present study shows for the first time, that prenatal THC exposure can profoundly alter the developing mesolimbic lipidome (Fig. 3), disrupting the bioavailability of multiple fatty acids throughout neurodevelopment that are necessary for normative cortical maturation, while inducing a plethora of enduring neurochemical aberrations.

The ECb system directly modulates mesolimbic DAergic projections from the VTA to the NAsh through CB1R activation (Tan et al., 2014; Wenzel and Cheer, 2018). Specifically, CB1R signaling modulates VTA DA release via inhibitory NAc originating GABAergic terminals (Tan et al., 2014; Wenzel and Cheer, 2018). As the mesolimbic pathway is implicated in anxiety and reward processing, alterations in VTA DAergic activity states paralleled with intra-NAc downregulation of D2R expression (Fig. 4), may be attributable to altered PCE-induced ECb signaling, selectively in males (Hernandez and Cheer, 2015; Wenzel and Cheer, 2018). In addition, the concomitant deficits in DHA and ARA fatty acid signaling are consistent with these mesolimbic aberrations, given their important functional roles in the normative regulation of DA/GLUT/GABAergic neurotransmission (Piomelli et al., 1991; Zimmer et al., 2002; Lafourcade et al., 2011). Interestingly, while D2R disruption and hyperactive VTA DAergic activity states were associated with anxiogenic effects, they neither altered reward processing (i.e., morphine reward sensitivity; Fig. 1*A*) nor sucrose preference behaviors (i.e., anhedonia), at least at the dose and protocols used in this study. Interestingly, we have previously reported that acute pharmacological alterations in GLUTergic transmission in the rat PFC (e.g., reducing NMDA receptor activation states) can strongly potentiate the rewarding properties of this subthreshold morphine conditioning dose (0.05 mg/kg, i.p.; Bishop et al., 2011; de Jaeger et al., 2013). Nevertheless, the present findings do not entirely preclude the possibility that sensitivity to other opioid doses and/or rewarding natural or drug stimuli may not be impacted by prenatal THC exposure (Fig. 1*G*). Additionally, our molecular analyses revealed potent dysregulation of selective GLUT/GABAergic molecular markers (Fig. 4). GLUT/GABAergic substrates are functionally involved in regulation of mesolimbic DAergic function and directly modulate intra-NAc DA neurotransmission (Rey et al., 2012; Covey and Yocky, 2021). Critically, CB1Rs are located primarily on GLUT axons and GABA presynaptic terminals in both the VTA and NAc (Rey et al., 2012; Guzman et al., 2021); suggesting a particular susceptibility of both systems to PCE which may result in the observed long-term dysregulation of the mesolimbic circuit.

Despite showing no behavioral abnormalities, female offspring displayed significant and persistent deficits in several GLUT/GABAergic signaling markers (Fig. 4). Interestingly, a critical neuronal sex difference we observed was the lack of VTA DAergic dysregulation in females (Fig. 2), consistent with previous studies (Traccis et al., 2021), and no corresponding modulation of DA D2R expression levels in the NAc (Fig. 4). One possibility is that VTA DAergic dysregulation in males serves as the primary driver of the observed behavioral abnormalities. Although females were resistant to VTA DA dysregulation (Fig. 2) and recovered sooner from lipidomic deficits (Fig. 3), PCE may lead to enduring neurochemical alterations in female offspring that were not sufficient to induce observable behavioral abnormalities, at least in the chosen assays. Alternatively, the observed molecular pathophenotypes observed in female brains may originate from other neural sources beyond the mesolimbic system (i.e., VTA-NAc), such as the cortex, amygdala, or hippocampus (Nestler and Lüscher, 2019). Future studies are required to further explore male versus female neuroanatomical trajectories in these phenotypic domains and the potential underlying protective mechanisms that appears to confer resilience to PCE-induced anxiogenic outcomes selectively in female offspring.

The profound lipidomic alterations observed in the present study would suggest THC-induced pathophysiological disturbances in mesolimbic synaptic integrity and function, especially given that normative perinatal ARA and DHA accumulation is required for optimal neurodevelopment and synaptogenesis (Basak et al., 2021). The observed ARA deficits were accompanied by altered downstream ARA signaling substrates, including adrenic acid (AA; Beasley et al., 2020), which was selectively disturbed in males only (Table 1). AA signaling is associated with regulating reelin expression and is reduced in schizophrenia patients, potentially because of dysregulated ARA-dependent upstream signaling (Messamore and Yao, 2016; Beasley et al., 2020). Importantly, proper neuronal migration and normative corticogenesis are contingent on ARA-dependent pathways that act in concert with reelin and are critical during the perinatal period of neurodevelopment (Messamore et al., 2010; Messamore and Yao, 2016). Furthermore, eicosatrienoic acid was dysregulated in males and females (Fig. 3). Critically, eicosatrienoic acid is an ARA-derived biologically active eicosanoid, functionally dependent on GLUTergic activity for formation, with their dysregulation being associated with schizophrenia and Alzheimer’s disease (Das, 2013; Chen et al., 2020). Thus, PCE appears to significantly alter ARA-dependent signaling pathways, which may in turn lead to long-term disruptions in normal cortico-striatal development.

Importantly, females appeared to be protected from the long-term lipidomic reductions observed in males. The observed recovery in fatty acid levels in females could potentially be explained by an estrogen dependent PUFA production pathway mediated by PPARα (Kitson et al., 2010; Wen-Ting et al., 2019). Indeed, estrogen receptors can activate PPARα-dependent lipid metabolism pathways to convert α-linolenic acid to DHA, potentially serving as an alternative compensatory pathway for the PCE-induced DHA deficits (Kitson et al., 2010; Wen-Ting et al., 2019). Support for this protective mechanism was suggested by our findings that only females (at PD120) exhibited significantly increased PPARα and ɣ1/2 NAc expression levels, both of which are involved in lipid metabolism (Walczak and Tontonoz, 2002; Kitson et al., 2010; Wen-Ting et al., 2019). Importantly, estrogen cycling is an important regulator of the DHA production pathway in females (Walczak and Tontonoz, 2002; Kitson et al., 2010). Given that female rats are still sexually immature at PD21 (van Weissenbruch et al., 2005), an alternative possibility is that, following puberty, increased estrogen production in female offspring may underlie the fatty acid normalization we observed at PD120 (Fig. 3; Table 1). For example, it is well established that estrogen receptors require DHA-rich lipid rafts for normal function, given their role in compartmentalizing cell signaling molecules (Kitson et al., 2010; Lauritzen et al., 2016). However, these receptors also allow for the recruitment of more DHA into the membrane (Kitson et al., 2010; Kim et al., 2019; Majou, 2021). Interestingly, female rats typically have greater DHA bioavailability than males to prepare for the cost of pregnancy, which may provide additional protection from the effects of THC on the developing female lipidome (van Weissenbruch et al., 2005; Kitson et al., 2010). Together, these mechanisms may provide a female-specific alternate DHA compensatory pathway unavailable to the developing male brain.

In addition, estrogen may account for the resistance to DAergic hyperactivity we observed in females (Fig. 2). For example, previous studies have demonstrated that estrogen receptor agonists can attenuate the effects of acute amphetamine on psychosis-like effects in female rats (Sbisa et al., 2018). More importantly, estrogen was found to be protective against the effects of prenatal amphetamine exposure on several measures of mesolimbic, intra-NAc DAergic sensitization in female versus male rats (Pennacchio et al., 2022). Thus, female behavioral and electrophysiological resiliency in adulthood may in part be because of these or other estrogen-related compensatory mechanisms of fatty acid production and/or resistance to DAergic sensitization that would normally be induced by THC exposure. Future studies are required to examine these potential female-specific protective mechanisms more precisely.

In summary, we report several novel and sex-dependent effects of prenatal THC exposure on the development of the mesolimbic system and associated behavioral, molecular, and fatty-acid-related biomarkers. These findings have several critical implications for the use of cannabis during pregnancy. First, the dose of THC dosage used here is moderate. Nevertheless, we observed multiple long-lasting pathophysiological effects on neurodevelopment even at these relatively low (i.e., 6%) maternal exposure concentrations (Gillies et al., 2020; Natale et al., 2020; Roncero et al., 2020; K Lee et al., 2021). Second, we report for the first time, severe long-term sex and age-dependent effects of prenatal cannabinoid exposure on the developing striatal lipidome. Interestingly, male offspring displayed remarkably greater levels of vulnerability to these risk factors suggesting potentially important neuroprotective factors remaining to be explored in the female brain. Finally, the present findings raise the possibility that interventions aimed at normalization of the neural lipidome with targeted fatty acid dietary interventions, may be a potential therapeutic target for prevention or possibly reversal of PCE-induced neuropathological outcomes.

## Synthesis

Reviewing Editor: Michael Michaelides, NIDA-NIH Decisions are customarily a result of the Reviewing Editor and the peer reviewers coming together and discussing their recommendations until a consensus is reached. When revisions are invited, a fact-based synthesis statement explaining their decision and outlining what is needed to prepare a revision will be listed below. The following reviewer(s) agreed to reveal their identity: NONE. Note: If this manuscript was transferred from JNeurosci and a decision was made to accept the manuscript without peer review, a brief statement to this effect will instead be what is listed below.

Reviewer 1

In the manuscript, the authors study a PCE animal model probing for difference between vehicle and exposed animals. The work in this manuscript is robust including both behavioral and biochemical measurements. The use of mass spectrometry imaging (MSI) is a novel approach to study spatial changes of lipids due to PCE. However, the authors should address several issues mostly pertaining to the MSI study.

1. The authors use MSI to study fatty acid changes in coronal rat brain sections. However, it is very difficult to study endogenous free fatty acids by mass spectrometry due to the fragmentation of more complex lipids such as phospholipids that can generate fatty acids. The authors should state this in the paper and especially in the Discussion section since the changes they see in fatty acids could be due to changes in more complex lipids and not free fatty acids.

2. In regards to the point above, did the authors conduct MSI studies in positive/negative ion mode in the range of the phospholipids. MSI is well-suited to study phospholipids where concerns of fragmentation or minimal when compared to free fatty acids. This result could be quite valuable as it would show if the changes in free fatty acids are seen across all phospholipid classes or localized to only a couple.

3. In the manuscript, the authors only show MSI images for DHA. Could the authors show additional images similar to Figure 3 for some of the other fatty acids in the Supplemental Data.

4. The authors should label the regions of interest (NAcc and NAsh) on the images in Figure 3A-D. I assume the area in red lines are the regions of interest that were used to generate the data in Figure 3F. It would be useful for the readers if the authors used a different color line for NAcc and NAsh regions of interest. Did the authors use both hemispheres of the coronal sections to generate the data in Figure3F.

Reviewer 2

Main comments:

In this study, Sarikahya et al., report the appearance of long-lasting alterations in anxiety-like behaviors following prenatal cannabis exposure (PCE) in rats. These adaptations were accompanied by neuronal, proteinic and lipidic changes throughout the mesolimbic system, suggesting a putative lipidome-related mechanism that could give rise to sex-specific effects of PCE. Most of the experiments are well designed, executed and reported. The Manuscript is elegantly written, and the topic, in lieu of the current surge in cannabis use during pregnancy, is of capital relevance. My only criticism is related to the characterization of reward-processing impairments in the PCE offspring, more comments below:

Major revisions：

• In the CPP experiment, VEH animals show a lack of preference towards the drug-paired compartment (Fig 1A). This result should preclude the Authors to conclude that PCE did not induce reward-processing alterations. It is true that the specific protocol employed (sub-threshold morphine dose) should not induce significant morphine CPP, but a positive control is always required to certify that the procedure worked. Without an effective dose of morphine, it is not possible to even conclude that the drug had significant central effects in this study.

As per the sucrose preference test, while it is true that it certainly informs about hedonic alterations, one cannot conclude that it totally excludes the presence of reward-processing changes. Primary food reinforcement is a reward response, but other core features of the reward processing, such as effort execution, approach, sustained engagement, or instrumental learning are left aside when using the sucrose preference test.

For these reasons, I would suggest the Authors to add a morphine dose that does induce CPP in VEH animals. If not possible, I would suggest the Authors to refer from concluding the absence of reward-processing effects of PCE and limit their interpretations to the very specific constructs evaluated by the sucrose preference test.

Minor revisions：

• Ln 67: while the use of cannabis during pregnancy is certainly on the rise, there is no epidemiological evidence of an “exponential” increase. I would refer from using this term.

• Ln 310-311: Instead of transition latency, open arm entries statistics should be reported.

• How do the Authors explain unaltered thigmotaxis in the PCE offspring when there is such a clear anxiety-like phenotype in other tests?

• A representative coronal section in Fig 3D suggests an important reduction of fatty acid concentration in PCE females at PD120. However, results indicate that there are no differences at this time point. Authors should choose a more representative image that does not have clear differences between these two groups.

• Please, delineate the specific ROIs used for NAcc and Nash in Figures 3A-D.

• In Figure 4F, all control bars seem to average above 100%. Please, check that the normalization was correctly calculated in such case.

• Ln 445: I would suggest replacing “associated” with “paralleled", given the lack of VTA DA activity measurements in behaving animals during the anxiety tests.
