# Supplementary figures and images for: Prenatal THC Exposure Induces Sex-Dependent Neuropsychiatric Endophenotypes in Offspring and Long-Term Disruptions in Fatty-Acid Signaling Pathways Directly in the Mesolimbic Circuitry
Source: eNeuro. 2022 Oct 7;9(5):ENEURO.0253-22.2022. doi: 10.1523/ENEURO.0253-22.2022 (PMC9557330; doi:10.1523/ENEURO.0253-22.2022)

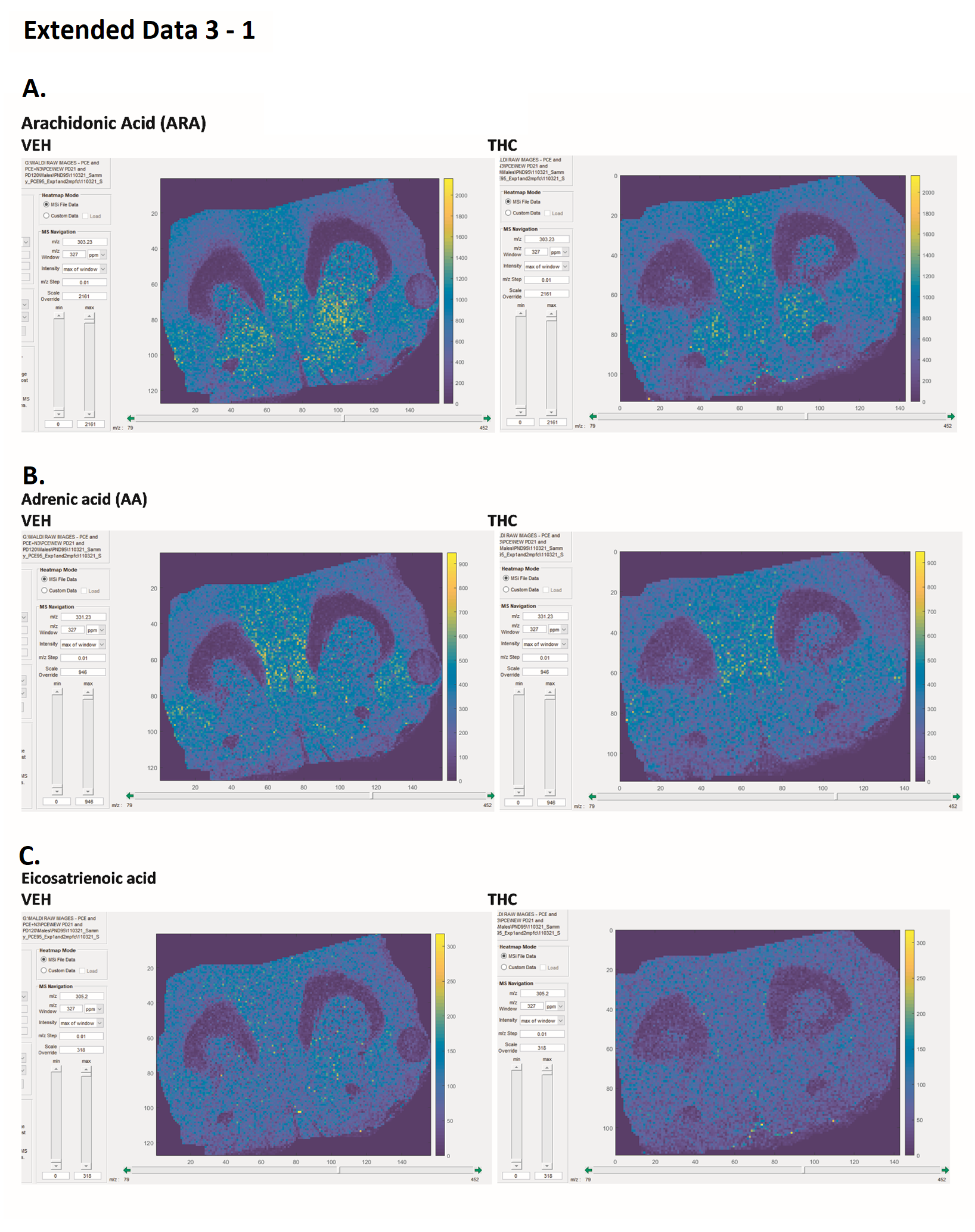

Supplement: Extended Data Figure 3-1 — A, Representative MALDI IMS image for arachidonic acid for PD120 male VEH versus THC progeny. B, Representative MALDI IMS images for adrenic acid for PD120 male VEH versus THC progeny. C, Representative MALDI IMS images for eicosatrienoic acid for PD120 male VEH versus THC progeny. Download Figure 3-1, TIF file. [file enu-eN-NWR-0253-22-s01.tif]

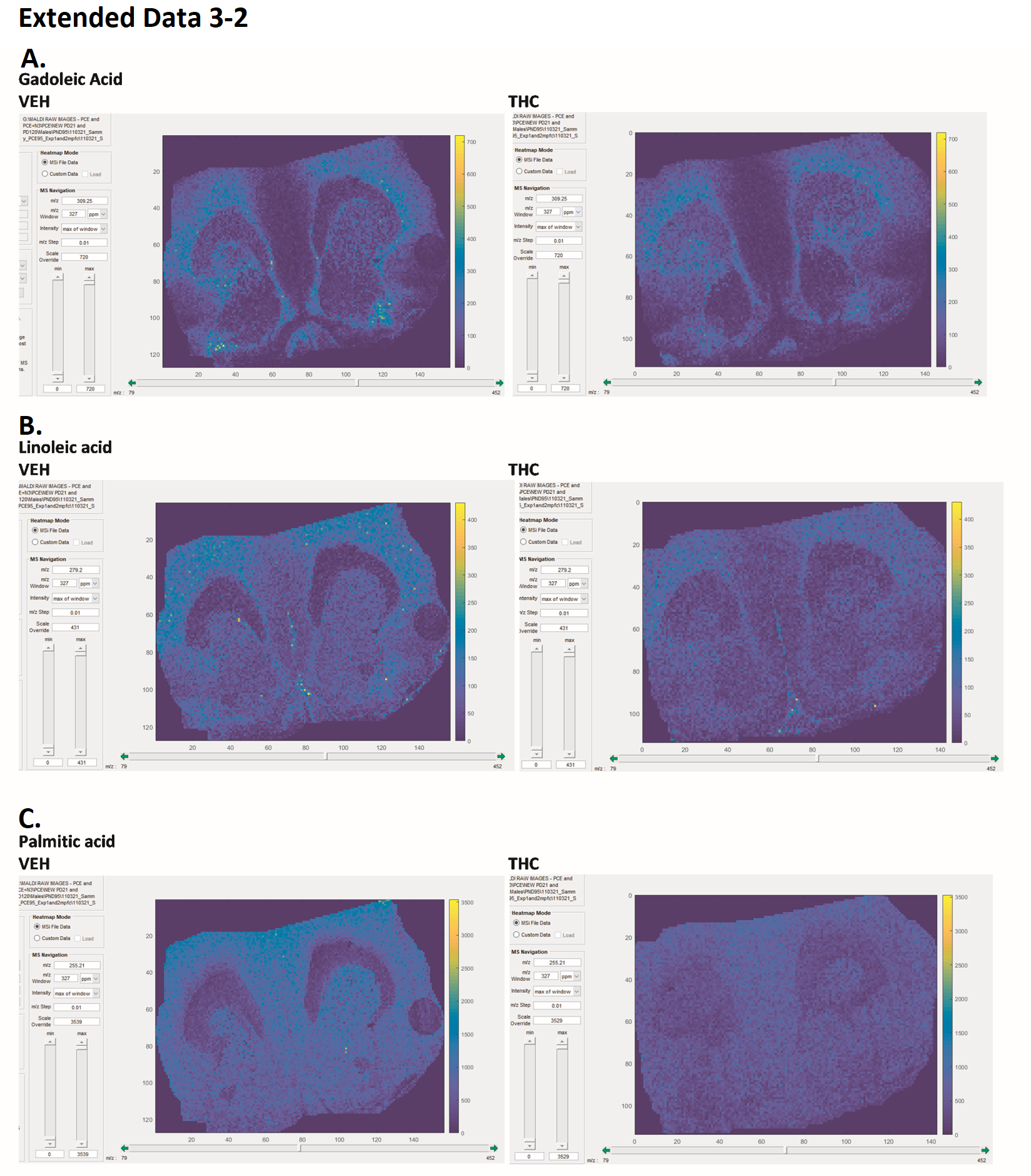

Supplement: Extended Data Figure 3-2 — A, Representative MALDI IMS image for gadoleic acid for PD120 male VEH versus THC progeny. B, Representative MALDI IMS images for linoleic acid for PD120 male VEH versus THC progeny. C, Representative MALDI IMS images for palmitic acid for PD120 male VEH versus THC progeny. Download Figure 3-2, TIF file. [file enu-eN-NWR-0253-22-s02.tif]

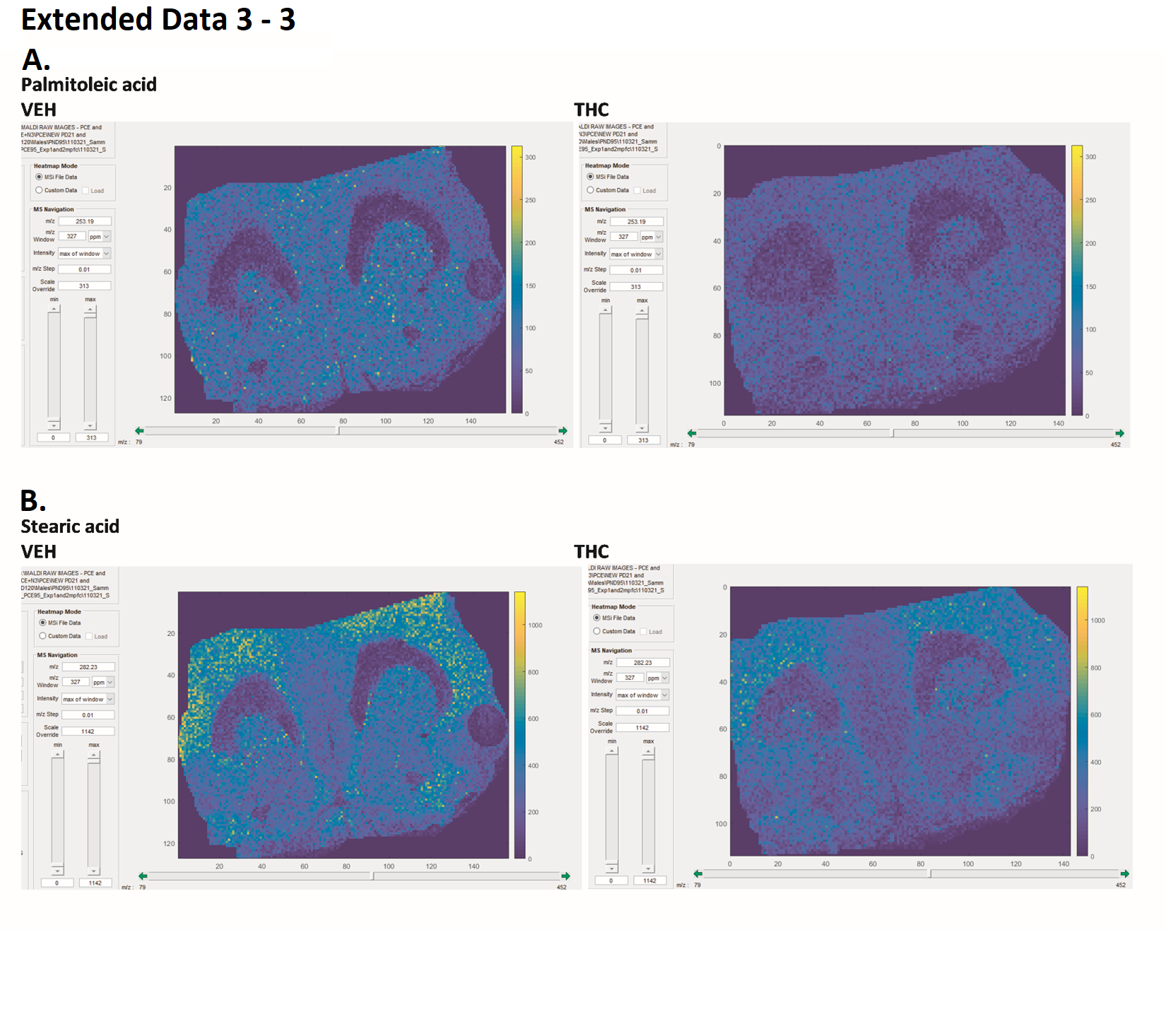

Supplement: Extended Data Figure 3-3 — A, Representative MALDI IMS image for palmitoleic acid for PD120 male VEH versus THC progeny. B, Representative MALDI IMS images for stearic acid for PD120 male VEH versus THC progeny. Download Figure 3-3, TIF file. [file enu-eN-NWR-0253-22-s03.tif]
